# Supplementary material for: The deployment of temporary nurses and its association with permanently-employed nurses’ outcomes in psychiatric hospitals: a secondary analysis
Source: PeerJ. 2023 Apr 28;11:e15300. doi: 10.7717/peerj.15300 (PMC10150716; doi:10.7717/peerj.15300)
Supplement: Supplemental Information 1 — N = number; SD = standard deviation; IQR = interquartile range. [file peerj-11-15300-s001.docx]

Supplementary 1

*Descriptive analysis of the unit sample (n = 79)*

| **Variables** | **N (%)** | **Mean (SD)** | **Median [IQR]** | **Range** |
| --- | --- | --- | --- | --- |
| Frequency of temporary nurses’ deployment |  |  |  |  |
| Occasionally or never | 59 (75%) |  |  |  |
| Frequently | 20 (25%) |  |  |  |
| Adjusted staffing (early shift) |  | 11.4 (1.1) | 11.3 [10.7, 11.9] | 8.8, 14.4 |

*Note*. N = number; SD = standard deviation; IQR = interquartile range.
