# Supplementary material for: The deployment of temporary nurses and its association with permanently-employed nurses’ outcomes in psychiatric hospitals: a secondary analysis
Source: PeerJ. 2023 Apr 28;11:e15300. doi: 10.7717/peerj.15300 (PMC10150716; doi:10.7717/peerj.15300)
Supplement: Supplemental Information 2 — N = number; SD = standard deviation; IQR = interquartile range. [file peerj-11-15300-s002.docx]

Supplementary 2

*Descriptive analysis of the nurse sample for the adjusted staffing model (n = 879)*

| **Variables** | **N (%)** | **Mean (SD)** | **Median [IQR]** | **Range** |
| --- | --- | --- | --- | --- |
| Patient-to-nurse ratio |  | 9.1 (5.1) | 8.0 [5.5, 10.5] | 0.1, 30.0 |
| Skill & grade mix in % |  | 71.6 (21.7) | 66.7 [50.0, 100.0] | 14.3, 100.0 |
| Shift |  |  |  |  |
| Early shift | 447 (51) |  |  |  |
| Late shift | 299 (34) |  |  |  |
| Night shift | 133 (15) |  |  |  |
| Somatic diagnoses ratio in % |  | 18.8 (24.8) | 10.0 [0.0, 25.0] | 0.0, 100.0 |
| Turnover ratio in % |  | 12.2 (17.4) | 5.9 [0.0, 17.0] | 0.0, 100.0 |

*Note*. N = number; SD = standard deviation; IQR = interquartile range.
