# Supplementary material for: The deployment of temporary nurses and its association with permanently-employed nurses’ outcomes in psychiatric hospitals: a secondary analysis
Source: PeerJ. 2023 Apr 28;11:e15300. doi: 10.7717/peerj.15300 (PMC10150716; doi:10.7717/peerj.15300)
Supplement: Supplemental Information 3 — CI = confidence interval; σ2 = residual variance; τ = rank correlation coefficient; ICC = Interclass correlation; N = number; R2 = R-squared; *p < 0.05; **p < 0.01; ***p < 0.001 [file peerj-11-15300-s003.docx]

Supplementary 3

*Adjusted staffing model with the frequency of temporary nurses’ deployment*

|  | **Patient-to-nurse ratio** | |
| --- | --- | --- |
| *Coefficient* | *Estimates* | *CI (95%)* |
| Intercept | 11.083 ^***^ | 9.681 – 12.485 |
| Skill & Grade Mix | -0.047 ^***^ | -0.063 – -0.031 |
| Late shift | 1.766 ^***^ | 1.028 – 2.503 |
| Night shift | 8.644 ^***^ | 7.682 – 9.607 |
| Somatic diagnoses ratio | -0.013 | -0.028 – 0.003 |
| Turnover ratio | -0.033 ^***^ | -0.052 – -0.014 |
| Frequent deployment of temporary | 0.692 | -0.389 – 1.774 |
| **Random Effects** | | |
| σ^2^ | 15.81 | |
| τ_00_ _unit_code_ | 2.19 | |
| ICC | 0.12 | |
| N _unit_code_ | 79 | |
| Observations | 598 | |
| Marginal R^2^ / Conditional R^2^ | 0.383 / 0.458 | |

*Note*. CI = confidence interval; σ^2^ = residual variance;

τ = rank correlation coefficient; ICC = Interclass correlation;

N = number; R^2^ = R-squared; * p < 0.05; ** p < 0.01; *** p < 0.001
