# Supplementary material for: The deployment of temporary nurses and its association with permanently-employed nurses’ outcomes in psychiatric hospitals: a secondary analysis
Source: PeerJ. 2023 Apr 28;11:e15300. doi: 10.7717/peerj.15300 (PMC10150716; doi:10.7717/peerj.15300)
Supplement: Supplemental Information 4 — CI = confidence interval; σ2 = residual variance; τ = rank correlation coefficient; ICC = Interclass correlation; R2 = R-squared; *p < 0.05; **p < 0.01; ***p < 0.001 [file peerj-11-15300-s004.docx]

Supplementary 4

*Final model without leadership and variables related to the four permanently-employed nurses’ outcomes*

|  | **Job satisfaction** | | **Burnout** | | **Intention to leave the organization** | | **Intention to leave the profession** | |
| --- | --- | --- | --- | --- | --- | --- | --- | --- |
| *Coefficient* | *Estimates* | *CI (95%)* | *Estimates* | *CI (95%)* | *Estimates* | *CI (95%)* | *Estimates* | *CI (95%)* |
| (Intercept) | 2.859 *** | 1.869 – 3.849 | 2.538 *** | 1.807 – 3.269 | 2.269 *** | 1.226 – 3.313 | 1.916 *** | 1.210 – 2.622 |
| Age | 0.007 * | 0.001 – 0.012 | -0.006 * | -0.011 – -0.001 | -0.017 *** | -0.023 – -0.011 | -0.011 *** | -0.016 – -0.005 |
| Sex [Male] | -0.013 | -0.157 – 0.130 | -0.032 | -0.157 – 0.093 | 0.049 | -0.110 – 0.207 | 0.045 | -0.103 – 0.194 |
| Employment percentage |  |  |  |  |  |  |  |  |
| *61%-95%* | 0.077 | -0.107 – 0.261 | 0.225 ^**^ | 0.064 – 0.385 | 0.079 | -0.124 – 0.282 | -0.055 | -0.245 – 0.135 |
| *96%-100%* | 0.015 | -0.177 – 0.207 | 0.162 | -0.005 – 0.329 | 0.090 | -0.122 – 0.302 | -0.083 | -0.280 – 0.115 |
| Adjusted staffing | 0.005 | -0.078 – 0.089 | 0.001 | -0.060 – 0.061 | 0.027 | -0.061 – 0.114 | 0.011 | -0.046 – 0.068 |
| Frequency of temporary nurses’ deployment [Frequently] | -0.243 ^*^ | -0.454 – -0.031 | 0.254 ^**^ | 0.098 – 0.411 | 0.243 ^*^ | 0.020 – 0.466 | 0.236 ^**^ | 0.083 – 0.388 |
| **Random Effects** |  |  |  |  |  |  |  |  |
| σ^2^ | 0.63 | | 0.49 | | 0.77 | | 0.71 | |
| τ_00_ | 0.09 _unit_code_ | | 0.03 _unit_code_ | | 0.09 _unit_code_ | | 0.00 _unit_code_ | |
| ICC | 0.12 | | 0.06 | | 0.10 | |  | |
| Marginal R^2^ / Conditional R^2^ | 0.028 / 0.146 | | 0.044 / 0.100 | | 0.066 / 0.162 | | 0.039 / NA | |

*Note*. CI = confidence interval; σ^2^ = residual variance; τ = rank correlation coefficient; ICC = Interclass correlation; R^2^ = R-squared; * p < 0.05; ** p < 0.01; *** p < 0.001
