# Supplementary material for: The deployment of temporary nurses and its association with permanently-employed nurses’ outcomes in psychiatric hospitals: a secondary analysis
Source: PeerJ. 2023 Apr 28;11:e15300. doi: 10.7717/peerj.15300 (PMC10150716; doi:10.7717/peerj.15300)
Supplement: Supplemental Information 5 — CI = confidence interval; σ2 = residual variance; τ = rank correlation coefficient; ICC = Interclass correlation; R2 = R-squared; *p < 0.05; **p < 0.01; ***p < 0.001 [file peerj-11-15300-s005.docx]

Supplementary 5

*Final model with leadership and variables related to the four permanently-employed nurses’ outcomes*

|  | **Job satisfaction** | | **Burnout** | | **Intention to leave the organization** | | **Intention to leave the profession** | |
| --- | --- | --- | --- | --- | --- | --- | --- | --- |
| *Coefficient* | *Estimates* | *CI (95%)* | *Estimates* | *CI (95%)* | *Estimates* | *CI (95%)* | *Estimates* | *CI (95%)* |
| (Intercept) | 0.803 ^*^ | 0.101 – 1.505 | 3.548 ^***^ | 2.832 – 4.264 | 3.927 ^***^ | 3.081 – 4.774 | 2.847 ^***^ | 2.124 – 3.570 |
| Age | 0.003 | -0.001 – 0.008 | -0.004 | -0.008 – 0.001 | -0.015 ^***^ | -0.021 – -0.009 | -0.009 ^**^ | -0.014 – -0.003 |
| Sex [Male] | 0.017 | -0.101 – 0.135 | -0.049 | -0.167 – 0.069 | 0.021 | -0.123 – 0.166 | 0.024 | -0.119 – 0.167 |
| Employment percentage |  |  |  |  |  |  |  |  |
| *61%-95%* | -0.054 | -0.205 – 0.097 | 0.286 ^***^ | 0.135 – 0.438 | 0.184 | -0.002 – 0.370 | 0.003 | -0.180 – 0.186 |
| *96%-100%* | -0.141 | -0.299 – 0.017 | 0.231 ^**^ | 0.073 – 0.389 | 0.210 ^*^ | 0.016 – 0.404 | -0.024 | -0.214 – 0.167 |
| Leadership | 0.768 ^***^ | 0.687 – 0.849 | -0.379 ^***^ | -0.460 – -0.297 | -0.609 ^***^ | -0.709 – -0.509 | -0.355 ^***^ | -0.451 – -0.260 |
| Adjusted staffing | -0.003 | -0.059 – 0.052 | 0.006 | -0.051 – 0.062 | 0.033 | -0.034 – 0.099 | 0.017 | -0.038 – 0.072 |
| Frequency of temporary nurses’ deployment [Frequently] | -0.100 | -0.244 – 0.044 | 0.189 ^*^ | 0.042 – 0.336 | 0.132 | -0.041 – 0.305 | 0.179 ^*^ | 0.031 – 0.326 |
| **Random Effects** |  |  |  |  |  |  |  |  |
| σ^2^ | 0.43 | | 0.43 | | 0.66 | | 0.66 | |
| τ_00_ | 0.02 _unit_code_ | | 0.03 _unit_code_ | | 0.03 _unit_code_ | | 0.00 _unit_code_ | |
| ICC | 0.05 | | 0.06 | | 0.04 | |  | |
| Marginal R^2^ / Conditional R^2^ | 0.377 / 0.407 | | 0.158 / 0.204 | | 0.247 / 0.278 | | 0.112 / NA | |

*Note*. CI = confidence interval; σ^2^ = residual variance; τ = rank correlation coefficient; ICC = Interclass correlation; R^2^ = R-squared; * p < 0.05; ** p < 0.01; *** p < 0.001
