# Supplementary material for: The deployment of temporary nurses and its association with permanently-employed nurses’ outcomes in psychiatric hospitals: a secondary analysis
Source: PeerJ. 2023 Apr 28;11:e15300. doi: 10.7717/peerj.15300 (PMC10150716; doi:10.7717/peerj.15300)
Supplement: Supplemental Information 6 — Note. n = number; SD = standard deviation; SMD = standardized mean difference. [file peerj-11-15300-s006.docx]

Supplementary 6

*Sensitivity analysis (n = 1012)*

| **Variables** | **Units without information on the deployment of temporary nurses** | **Units with information on the deployment of temporary nurses** | **SMD** |
| --- | --- | --- | --- |
| Overall n | 323 | 689 |  |
| Sex (n (%)) |  |  | 0.022 |
| Female | 218 (67.9) | 473 (69.0) |  |
| Male | 103 (32.1) | 213 (31.0) |  |
| Age (mean (SD)) | 41.6 (12.4) | 41.0 (12.3) | 0.051 |
| Employment percentage (n (%)) |  |  | 0.036 |
| <60% | 52 (16.5) | 112 (16.6) |  |
| 61% - 95% | 146 (46.3) | 301 (44.7) |  |
| 96% - 100% | 117 (37.1) | 261 (38.7) |  |

*Note*. n = number; SD = standard deviation; SMD = standardized mean difference.
